# Long-term Effects of 3-Month Home-Based Cardiac Rehabilitation Using Information and Communication Technology for Heart Failure with Physical Frailty

**DOI:** 10.1016/j.cjco.2025.07.012

**Published:** 2025-07-30

**Authors:** Yuta Nagatomi, Tomomi Ide, Takeo Fujino, Takeshi Tohyama, Tae Higuchi, Tomoyuki Nezu, Takuya Nagata, Toru Hashimoto, Shouji Matsushima, Keisuke Shinohara, Tomiko Yokoyama, Masataka Ikeda, Shintaro Kinugawa, Hiroyuki Tsutsui, Kohtaro Abe

**Affiliations:** a Department of Rehabilitation Medicine, Kyushu University Hospital, Fukuoka, Japan; b Department of Cardiovascular Medicine, Faculty of Medical Sciences, Kyushu University, Fukuoka, Japan; c Department of Advanced Cardiopulmonary Failure, Faculty of Medical Sciences, Kyushu University, Fukuoka, Japan; d Department of Nutrition, Kyushu University Hospital, Fukuoka, Japan; e Department of Cardiovascular Medicine, NHO Fukuoka National Hospital, Fukuoka, Japan; f School of Medicine and Graduate School, International University of Health and Welfare, Fukuoka, Japan

**Keywords:** Cardiac rehabilitation, exercise tolerance, physical frailty, heart failure, home rehabilitation, telemedicine

## Abstract

**Background:**

Information and communication technology (ICT)-supported home-based cardiac rehabilitation (HBCR) has gained prominence because of its potential advantages, including improved patient engagement. However, the long-term effects on patients with heart failure (HF) and physical frailty are unclear. The aim of this study was to determine the effects of HBCR on patients with HF and physical frailty 12 months after the HBCR intervention.

**Methods:**

This single-centre, single-arm intervention trial included 30 outpatients with chronic HF and physical frailty or pre-frailty. Participants received a comprehensive ICT-based HBCR intervention, including disease management, exercise, and nutritional guidance for 3 months, followed by a 12-month period of ICT-supported self-management without professional guidance. The primary outcome was the change in 6-minute walking distance (6MWD).

**Results:**

The 6MWD of the patients significantly improved at 3 months, compared with baseline (395.8 ± 16.2 metres [95% confidence interval (CI): 363.0-428.6] vs 445.1 ± 16.3 metres [95% CI, 412.0-478.2]; *P* < 0.01), but it decreased at 15 months, compared with 3 months (417.7 ± 16.3 metres [95% CI: 384.6-450.8]; *P* = 0.04). The frailty score also decreased at the 3-month vs the 15-month timepoint. Patients who continued to exercise at 15 months showed sustained improvement in 6MWD.

**Conclusions:**

At 12 months after the intervention, the initial improvements in exercise tolerance and frailty were not maintained in the overall cohort. The ICT-supported self-management approach used in this study was insufficient to promote sustained behavioural change over the long term.

## Résumé

**Contexte:**

La réadaptation cardiaque à domicile (RCD) assistée par les technologies de l'information et de la communication (TIC) a gagné en importance en raison de ses avantages potentiels, notamment l'amélioration de l'engagement des patients. Cependant, ses effets à long terme sur les patients atteints d'insuffisance cardiaque (IC) et de fragilité physique ne sont pas clairs. L'objectif de cette étude était de déterminer les effets de la RCD sur les patients atteints d'IC et de fragilité physique 12 mois après l'intervention de RCD.

**Méthodologie:**

Cet essai interventionnel monocentrique à bras unique a inclus 30 patients ambulatoires atteints d'IC chronique et présentant une fragilité physique ou une préfragilité. Les participants ont bénéficié d'une intervention de RCD complète basée sur les TIC, comprenant la prise en charge de la maladie, des exercices physiques et des conseils nutritionnels pendant 3 mois, suivie d'une période de 12 mois d'autogestion assistée par les TIC sans accompagnement professionnel. Le critère d'évaluation principal était l’évolution de la distance parcourue au test de marche de six minutes (DM6M).

**Résultats:**

La DM6M des patients s'est considérablement améliorée à 3 mois par rapport à la valeur de référence (395,8 ± 16,2 m [intervalle de confiance (IC) à 95 %, 363,0 – 428,6] contre 445,1 ± 16,3 m [IC à 95 %, 412,0 – 478,2] ; *p* < 0,01), mais a diminué à 15 mois par rapport à 3 mois (417,7 ± 16,3 m [IC à 95 % : 384,6 – 450,8] ; *p* = 0,04). Le score de fragilité a également diminué entre 3 et 15 mois. Les patients qui ont continué à faire de l'exercice à 15 mois ont montré une amélioration durable de la DM6M.

**Conclusions:**

Douze mois après l'intervention de RCD, les améliorations initiales en matière de tolérance à l'exercice et de fragilité n'ont pas été maintenues dans l'ensemble de la cohorte. L'approche d'autogestion soutenue par les TIC utilisée dans cette étude s'est avérée insuffisante pour favoriser un changement de comportement durable à long terme.

Cardiac rehabilitation (CR) serves as a vital secondary prevention measure, offering numerous advantages, such as improving exercise capacity, enhancing health-related quality of life, and reducing hospitalization due to heart failure (HF).^1^^,^^2^ However, challenges such as transportation issues, lack of motivation, and financial concerns discourage patients from continuing CR, resulting in low adherence rates globally.^3^^,^^4^ In addition, adherence to exercise drops significantly after CR ends, with studies reporting rates below 8% at 1 year,^5^ suggesting that the formation of an exercise habit remains a major challenge.

Patients with HF and physical frailty, a group at high risk of functional decline and frequent rehospitalizations, require sustained support to maintain their exercise capacity.^6^^,^^7^ Home-based CR (HBCR) programs, involving exercise interventions guided by healthcare professionals, improve the exercise capacity of patients with HF and are associated with a higher level of adherence compared to the level with center-based CR.^8^^,^^9^ Recent advancements in information and communication technology (ICT)—including wearable devices and smartphone-based applications—have enabled CR delivery in remote or underserved settings.^10^, ^11^, ^12^ In our previous Home-Rehab study,^13^ we demonstrated that a 3-month comprehensive HBCR program using ICT and multidisciplinary support significantly improved 6-minute walking distance (6MWD) in patients with HF and physical frailty. Although long-term improvements in exercise tolerance have been demonstrated when structured professional support is maintained,^14^ the effects following the cessation of such support remain unclear.^15^ Although the use of ICT has shown promise in promoting adherence during the maintenance phase of CR,^16^^,^^17^ evidence supporting its long-term effectiveness—particularly in patients with HF and physical frailty—remains limited. Whether ICT-supported self-management can sustain exercise tolerance and establish long-term exercise habits is an important clinical question in real-world settings.

Therefore, the present study aimed to evaluate whether exercise tolerance could be maintained at 12 months following the initial 3-month HBCR intervention, during which patients continued self-monitoring with ICT tools but received no further structured rehabilitation. This follow-up study addresses a critical gap in understanding the sustainability of ICT-based rehabilitation strategies for patients with HF and physical frailty in real-world settings.

## Materials and Methods

### Study design and participants

This study evaluated a single-centre, single-arm intervention involving outpatients with chronic HF and physical frailty or pre-frailty. The data from an open-label, randomized control trial conducted from April to November 2020, as part of our previous Home-Rehab study,^13^ were utilized in this study. The patients in the control group who participated in the Home-Rehab study underwent the same intervention as those in the intervention group, but with a delayed start. The intervention lasted for 3 months, followed by a 12-month follow-up. All patients were encouraged to use Fitbit (Inspire HR, Miami, FL) for the entire duration of the study. The study was conducted at Kyushu University Hospital, located in Fukuoka, Japan.

The inclusion and exclusion criteria used for patient selection have been described previously.^13^ Briefly, patients with chronic HF and physical frailty or a pre-frailty stage were included. Patients with chronic HF are those with New York Heart Association class II or III. Patients with physical frailty were screened using the Japanese Cardiovascular Health Study (J-CHS) Scale,^18^ which was developed by modifying the original Cardiovascular Health Study criteria. The data for each patient were managed using Research Electronic Data Capture (REDCap), an electronic data-capture system.

### Comprehensive CR program

The comprehensive HBCR program using ICT was detailed in a previous report.^13^ Briefly, the program provided remote disease management, individualized exercise instruction, and nutritional guidance through use of the Fitbit device and application. The patients’ own step counts and pulse rates were monitored remotely, and the CR team—consisting of physical therapists, dietitians, nurses, and cardiologists—offered weekly feedback and tailored support.

After completing the 3-month HBCR program, patients were encouraged to maintain their lifestyle habits through self-management using the Fitbit device and application. During the 12-month self-management period, access to educational pamphlets and instructional exercise videos was provided to support continued self-management; however, no supervised rehabilitation sessions were offered. Patients continued to receive standard outpatient care every 2-3 months.

### Data collection

Data were collected at baseline, 3 months (after the intervention), and 15 months (12 months after the intervention had ended). The primary outcome was the change in 6MWD. The 6-minute walking test was performed in accordance with the American Thoracic Society protocol.^19^ The secondary outcomes were exercise adherence, isometric knee extension strength, brain natriuretic peptide (BNP) level, Kansas City Cardiomyopathy Questionnaire (KCCQ) scores, number of steps, and J-CHS scale. Exercise adherence was determined using a questionnaire regarding the frequency, duration, type, and number of exercises undertaken in the last 2 weeks during a 12-month observation follow-up period. Isometric knee extension strength was measured using a COMBIT CB-2 (Minato Medical Science, Osaka, Japan) to determine the maximal isometric knee extension strength (Nm) at 60° of knee flexion, and the weight ratio (kgf/kg) was calculated. Isometric knee extension strength was measured twice on each side, and the maximum value was recorded. Plasma BNP levels were measured by conducting blood chemical tests.^20^ Health-related quality of life was assessed using the KCCQ.^21^ The number of steps was measured by the Fitbit application, and the mean number of steps per day was calculated. Physical frailty was evaluated using the J-CHS.^18^

### Statistical analysis

Patient characteristics are reported as means ± standard deviations, or percentages, and the outcomes are reported as least-squares mean estimates at each time point, along with their corresponding standard errors and 95% confidence intervals (CIs). The primary and secondary outcomes were analyzed using mixed-effects models for repeated measures (MMRMs). The statistical modelling of the MMRM was conducted using a linear mixed-effect model with restricted maximum likelihood estimation. Multiple comparisons were performed using Tukey’s honest significant difference test within the MMRM framework. After 12 months of the follow-up period, the 6MWD, knee extension muscle strength, and number of steps were compared using MMRM between the 2 groups according to exercise adherence. Differences in 3-month characteristics were analyzed using the Wilcoxon rank-sum test for continuous variables and the χ^2^ test for categorical variables. All statistical tests were 2-tailed, and statistical significance was set at *P* < 0.05. All analyses were performed using JMP Pro 16 software and the SAS statistical package (SAS Institute, Cary, NC).

### Ethical considerations

This study was approved by the Ethics Committee of Kyushu University Hospital (Approval no. 20192031-1, 20192031-2, 21022-00), and written informed consent was obtained from all patients. The protocol was registered in the University Hospital Medical Information Network (Home-Rehab study; registration number UMIN000040136) before patient inclusion.

## Results

### Changes in exercise tolerance and muscle strength

**Figure 1:**
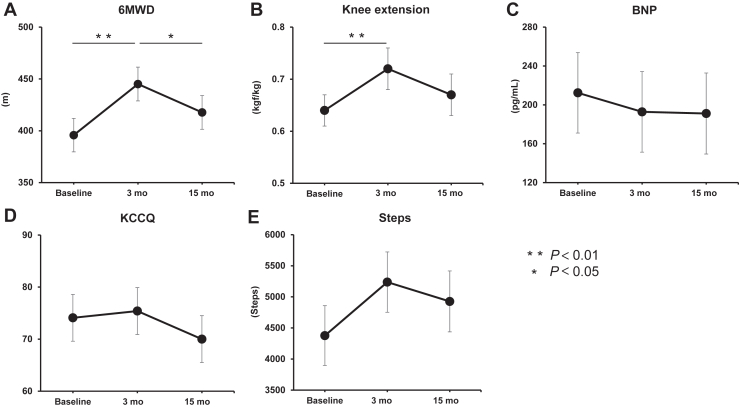
Outcomes of the home-based cardiac rehabilitation intervention. The changes in (**A**) 6-minute walk distance (6MWD), (**B**) knee extension strength; (**C**) brain natriuretic peptide (BNP) level; (**D**) Kansas City Cardiomyopathy Questionnaire (KCCQ) score, and (**E**) average of steps, at baseline, 3 months, and 15 months, are shown. The line chart shows the estimated least-squares means, and the error bars are shown as standard errors.

Of the 30 patients who participated in this study, 28 were assessed at the end of the study period (Supplemental Fig. S1). Two patients were lost to follow-up—one due to stroke, and the other due to being followed up at another hospital. Table 1 shows the patient characteristics after the 3-month intervention. The primary outcome, the 6MWD of the patients, significantly improved at 3 months compared with baseline (395.8 ± 16.2 metres (95% CI: 363.0-428.6) vs 445.1 ± 16.3 metres (95% CI: 412.0-478.2]; *P* < 0.01), but it decreased at 15 months, compared with 3 months (417.7 ± 16.3 metres [95% CI: 384.6-450.8]; *P* = 0.04; Fig. 1A). Among the secondary outcomes, knee extension muscle strength significantly improved at 3 months, compared with baseline (0.64 ± 0.03 kgf/kg [95% CI: 0.57-0.71] vs 0.72 ± 0.04 kgf/kg [95% CI: 0.65-0.79]; *P* < 0.01), but it decreased at 15 months, compared with 3 months (0.67 ± 0.04 kgf/kg [95% CI: 0.60-0.74]; *P* = 0.08; Fig. 1B). The other secondary outcomes, including BNP level, KCCQ score, and number of steps, were not significantly different at either 3 or 15 months (Fig. 1, C-E).

**Table 1:** Characteristics of the enrolled patients after a 3-month intervention

Characteristic	n = 30
Age, y	64.1 ± 10.0
Male	16 (53)
Body mass index, kg/m^2^	20.7 ± 2.5
NYHA class	
Ⅱ	19 (63)
Ⅲ	11 (37)
Physical frailty (J-CHS)	
Robust	12 (40)
Pre-frailty	16 (53)
Frailty	1 (3)
No data	1 (3)
Etiologies of heart failure	
Ischemic heart disease	4 (13)
Nonischemic heart disease	26 (87)
Medical history	
PCI	3 (10)
Valvular surgery	3 (10)
PMI and/or ICD and/or CRT-P and/or CRT-D	17 (57)
LVAD	4 (13)
Comorbidities	
Hypertension	6 (20)
Diabetes mellitus	5 (17)
Hyperlipidemia	6 (20)
Chronic kidney disease	7 (23)
Atrial fibrillation	3 (10)
Echocardiography	
LVEF, %	42.2 ± 17.4
LVEF < 40%	14 (47)
Medication	
ACE-I and/or ARB	23 (77)
Beta-blocker	29 (97)
MRA	18 (60)
Loop diuretic	10 (33)
Inotropic agent	7 (23)
Statin	8 (27)

Data are expressed as mean ± standard deviation or n (%).

ACE-I, angiotensin-converting enzyme inhibitor; ARB, angiotensin II receptor blocker; CRT-D, cardiac resynchronization therapy-defibrillator; CRT-P, cardiac resynchronization therapy-pacemaker; ICD, implantable cardiac defibrillator; J-CHS, Japanese version of the Cardiovascular Health Study Criteria; LVAD, left ventricular assist device; LVEF, left ventricular ejection fraction; MRA, mineralocorticoid receptor antagonist; NYHA, New York Heart Association; PCI, percutaneous coronary intervention; PMI, pacemaker implantation.

### Changes in physical frailty score

**Figure 2:**
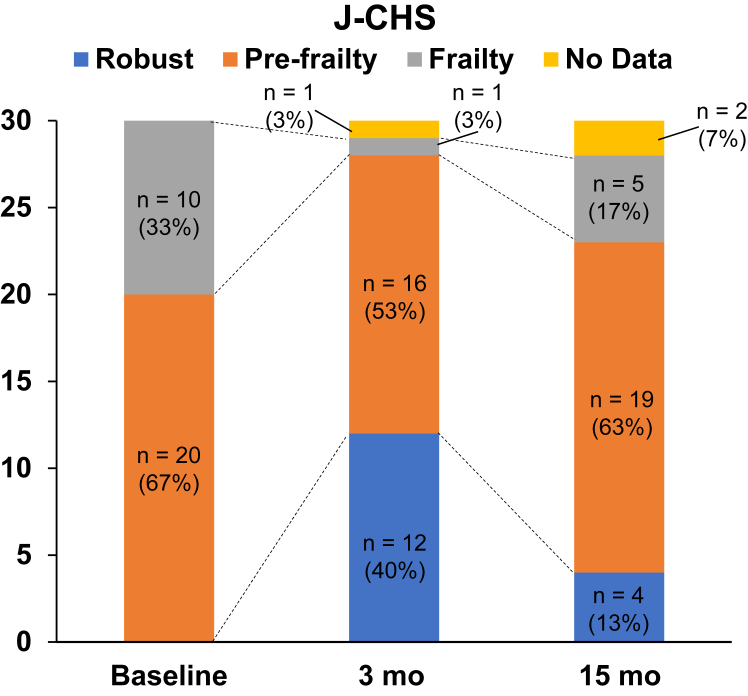
Changes in the frailty scale. The changes in the Japanese Cardiovascular Health Study (J-CHS) scale score, at baseline vs 3 months, vs 15 months, are shown.

The changes in the J-CHS score are shown in Figure 2. After 3 months of intervention, the number of patients in the frailty and pre-frailty stages decreased, and the number of patients in the robust stage increased. At 3 months, 1, 16, and 12 patients—3%. 53%. and 40%—were in the frailty, pre-frailty, and robust stages, respectively. However, at 15 months, the percentage of patients in the frailty and pre-frailty stages increased from 3% to 17%, and from 53% to 63%, respectively. The percentage of patients in the robust stage decreased from 40% to 13% (Fig. 2).

### Exercise adherence

The level of exercise adherence of the patients in the last 2 weeks of the follow-up period is shown in Supplemental Table S1. About half of patients continued to exercise ≥ 3 times a week (46.5%, 13 of 28), and some continued to exercise 3 times a week and for > 30 minutes (25%, 7 of 28). Walking was the most frequently reported type of exercise (67%, 14 of 21), and strength training was the least common (24%, 5 of 21). Moreover, most patients performed only one type of exercise (62%, 13 of 21). The number of patients who exercised ≥ 3 times a week increased from 10% at baseline to 76% and 46.5% at 3 and 15 months, respectively.

**Figure 3:**
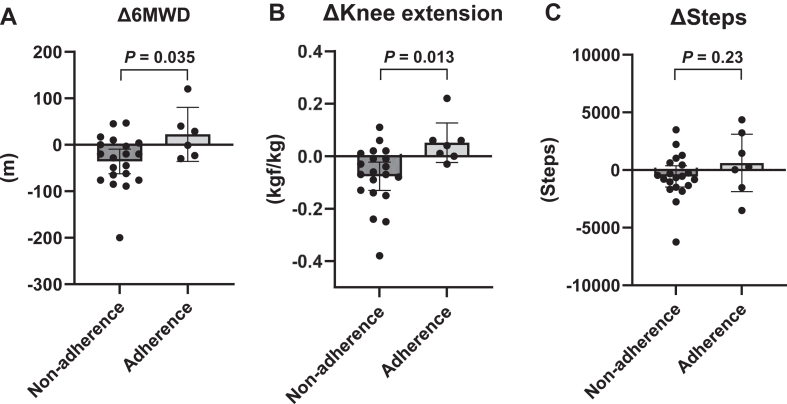
Comparison of the Adherence vs Non-adherence groups. Changes in the (**A**) 6-minute walking distance (6MWD), (**B**) knee extension, and (**C**) average number of steps, from 3 to 15 months in the Adherence and Non-adherence groups are shown.

At 15 months, the 7 patients (25%) who continued to exercise ≥ 3 times a week for ≥ 30 minutes per session were included in the Adherence group, and the remaining 21 patients (75%) who exercised l< 3 times a week or < 30 minutes per session were in the Non-adherence group. The changes in 6MWD, knee extension muscle strength, and the number of steps from 3 to 15 months in each group are shown in Figure 3. The 6MWD was significantly decreased in the Non-adherence group, and it was maintained or even increased in the Adherence group (Non-adherence group: –35.8 [95% CI: –61.6 to –9.9] metres; Adherence group: + 22.5 [95% CI: –24.8 to 69.8] metres, *P* = 0.035; Fig. 3A). Knee extension muscle strength was also significantly decreased in the Non-adherence group and was maintained in the Adherence group (Non-adherence group: –0.08 [95% CI: –0.13 to –0.03] metres; Adherence group: + 0.05 [95% CI: –0.03 to 0.14] kgf/kg, *P* = 0.013; Fig. 3B). No difference occurred in the number of steps for the Non-adherence vs Adherence groups (Non-adherence group: –556 [95% CI: –1556 to 444] steps; Adherence group: + 614 [95% CI: –1077 to 2305] steps, *P* = 0.23; Fig. 3C). The Adherence group had a higher proportion of male participants (86%) than the Non-adherence group (38%), but the difference was not statistically significant (*P* = 0.08; Supplemental Table S2). No other significant differences were observed in the 3-month characteristics between the Adherence and Non-adherence groups (Supplemental Table S2).

### Adverse events

No patients died during the study period, and 4 patients were rehospitalized for the following cardiovascular events during the 15-month period: HF (n = 1); arrhythmia (n = 1); stroke (n = 1); and arteriosclerosis obliterans (n = 1). Meanwhile, 8 patients were rehospitalized for noncardiovascular reasons, including fractures (n = 2), liver function abnormalities (n = 2), pneumonia (n = 1), dehydration (n = 1), electrolyte abnormalities (n = 1), and cholecystitis (n = 1; Supplemental Table S3).

## Discussion

In this single-arm interventional trial, we investigated whether the initial benefits observed after a 3-month ICT-supported HBCR program in patients with HF and physical frailty could be sustained during a subsequent 12-month self-management period. Although the structured intervention led to temporary improvements in exercise tolerance and frailty status, these benefits were not maintained at 12 months. This finding highlights the potential limitations of unsupervised, ICT-based self-management in this population. This study is the first to investigate the long-term effect of ICT-based HBCR, especially for patients with HF and frailty, who are high-risk patients with poor clinical outcomes.

Although several studies have demonstrated the long-term benefits of telerehabilitation in HF patients,^10^^,^^22^^,^^23^ few have focused on those with physical frailty. For example, a subanalysis of the HF-ACTION (Heart Failure: A Controlled Trial Investigating Outcomes of Exercise Training) trial showed that continued structured support led to sustained improvements in function among frail participants.^24^ Many of the studies that reported long-term improvements employed ongoing professional support or caregiver-involved interventions to promote behavioural change.^10^^,^^22^ In contrast, our study adopted a self-management approach after the 3-month intervention. The limited sustainability of benefits observed in this study suggests that the 3-month intervention period may have been insufficient, or that ICT-supported self-monitoring without continued professional involvement may not be sufficient to maintain behavioural changes in this vulnerable population.

In this study, some patients maintained exercise habits using wearable devices and self-monitoring. These tools have been shown to enhance physical activity and exercise tolerance,^25^ and mobile applications also are known to promote participation in CR.^26^ To explore potential factors influencing adherence, we compared 3-month characteristics between the Adherence and Non-adherence groups; however, no statistically significant differences were found. This result may be due to the limited sample size, making it difficult to identify distinct predictors of sustained adherence. Moreover, findings from the Pedometer-Based Walking Intervention in Patients With Chronic Heart Failure With Reduced Ejection Fraction (WATCHFUL) trial suggest that self-monitoring alone—even when combined with remote counselling—may not improve long-term outcomes, possibly due to insufficient exercise intensity.^27^ These results align with our findings and indicate that additional strategies, such as motivational support and structured follow-up, are necessary to sustain adherence and effectiveness.

During the HBCR phase, weekly goal-setting strategies were used to support motivation, but most patients were unable to maintain exercise frequency or intensity during the self-management phase. The Effect of Gamification, Financial Incentives, or Both to Increase Physical Activity Among Patients at High Risk of Cardiovascular Events (BE ACTIVE) trial recently demonstrated that behavioural economics approaches, such as gamification and financial incentives, can promote sustained physical activity after the intervention.^28^ These findings suggest that for patients with HF and frailty, combining ICT-based support with motivational strategies grounded in behavioural science may be necessary to ensure long-term exercise adherence and maximize rehabilitation benefits.

During the 12-month follow-up period, only one hospitalization for HF occurred, and no deaths occurred among the 30 patients. This low event rate suggests that the study population represented a relatively low-risk group. Most of the participants were relatively younger patients with stable clinical conditions who were managed in an outpatient setting. Although this favourable outcome may be partially attributable to the effects of the HBCR program using ICT, we cannot infer causality, and further studies with larger sample sizes and higher-risk populations are warranted to confirm the long-term benefits of such interventions.

### Limitations of the study

This study has several limitations. First, this was a single-arm study without a control group. Because this study enrolled all participants of our previous Home-Rehab study, we did not perform a sample size calculation in advance. Although we conducted a post hoc power analysis to assess the long-term effects of the HBCR intervention, the findings should be interpreted with caution and should be validated in larger randomized controlled trials. Second, the study population was relatively young. Further research is needed to evaluate the feasibility and effectiveness of ICT-based HBCR in elderly individuals who may have difficulty using digital technologies. Third, although all participants were patients with HF and physical frailty, the severity of HF varied. Due to the limited sample size, we did not perform the subgroup analysis using HF severity. Also, we did not perform subgroup or multivariate analyses using age, depression, and HF rehospitalization. Fourth, this study included several exclusion criteria to ensure safety in an unsupervised setting, such as locomotor disorders, cognitive decline, and advanced chronic kidney disease. Therefore, the findings may not be fully generalizable to the broader population of patients with HF encountered in routine clinical practice. Future research should include a broader and more diverse cohort and should consider stratified analyses to better capture subgroup-specific safety and efficacy. Finally, patients were given a recommendation to maintain physical activity with appropriate intensity after the intervention; however, we have no information on whether those patients perform adequate-intensity exercise . This lack of information also makes it difficult to ascertain the factors influencing the long-term outcomes of the intervention.

### Conclusions

This follow-up study found that the initial improvements in exercise tolerance and physical frailty status observed after a 3-month ICT-based HBCR program were not sustained at 12 months in patients with HF and physical frailty. Further research is required to develop consistent remote management for patients with HF that motivates them to continue making behavioural changes to increase their physical activity and exercise habits.

## Supplementary Material

Supplemantary materials
