# Supplementary material for: Long-term Effects of 3-Month Home-Based Cardiac Rehabilitation Using Information and Communication Technology for Heart Failure with Physical Frailty
Source: CJC Open. 2025 Jul 30;7(10):1390–7. doi: 10.1016/j.cjco.2025.07.012 (PMC12572893; doi:10.1016/j.cjco.2025.07.012)
Supplement: Supplemantary materials [file mmc1.pdf]

Supplemental Materials

Supplemental Table S1. Exercise adherence in the last 2 weeks of the follow-up period.

	n (%)
Frequency (n = 28)	
No exercise	7 (25)
1-2 times/week	8 (28.5)
3-4 times/week	8 (28.5)
≥ 5 times/week	5 (18)
Duration (n = 21)	
< 15 minutes	10 (48)
15 - 29 minutes	4 (19)
30 - 44 minutes	3 (14)
≥ 45 minutes	4 (19)
Type (n = 21)	
Walking	14 (67)
Stretching	7 (33)
Balance	7 (33)
Strength training	5 (24)
Number of exercises (n = 21)	
Only 1	13 (62)
Combination of 2	4 (19)
Combination of 3	3 (14)
Combination of 4	1 (5)

Data are expressed as n (%).

Supplemental Table S2. Comparison of 3-month characteristics between the Adherence and Non-adherence groups.

	Adherence (n=7)	Non-adherence (n=21)	P-value
Age, years	65.6 ± 8.5	64.0 ± 10.9	0.98
Male, n (%)	6 (86)	8 (38)	0.08
Body mass index, kg/m^2	21.0 ± 2.6	20.8 ± 2.5	0.98
NYHA class, n (%)			0.67
II	4 (57)	14 (67)	
III	3 (43)	7 (33)	
Physical Frailty (J-CHS), n (%)			0.83
Robust	3 (43)	9 (43)	
Pre-frailty	4 (57)	11 (52)	
Frailty	0 (0)	1 (5)	
Etiologies of heart failure, n (%)			1.00
Ischemic heart disease	1 (14)	3 (14)	
Non-ischemic heart disease	6 (86)	18 (86)	
Echocardiography			
LVEF, %	33.5 ± 15.4	46.3 ± 17.4	0.09
LVEF < 40%, n (%)	5 (71)	9 (43)	0.38
6MWD, m	460.0 ± 64.5	451.5 ± 92.9	0.81
Knee extension, kgf/kg	0.75 ± 0.2	0.69 ± 0.2	0.26
BNP, pg/mL	218.4 ± 189.5	185.3 ± 260.6	0.41
KCCQ	70.6 ± 23.3	81.1 ± 20.5	0.13
Steps, steps	6640 ± 3445	4864 ± 2098	0.23

Data are expressed as mean ± standard deviation and n (%).

NYHA, New York Heart Association; J-CHS, Japanese version of the Cardiovascular Health Study Criteria; LVEF, left ventricular ejection fraction; 6MWD, 6-minute walking distance; BNP, brain natriuretic peptide; KCCQ, Kansas city Cardiomyopathy Questionnaire.

Supplemental Table S3. Adverse events.

	n (%)
Cardiovascular events (n = 4)	
Heart failure	1 (4)
Arrhythmia	1 (4)
Stroke	1 (4)
Arteriosclerosis obliterans	1 (4)
Non-cardiovascular events (n = 8)	
Fractures	2 (7)
Liver function abnormalities	2 (7)
Pneumonia	1 (4)
Dehydration	1 (4)
Electrolyte abnormalities	1 (4)
Cholecystitis	1 (4)

Data are expressed as n (%).

Supplemental Figure S1. Study design diagram.

Flow chart of patients' selection process in this study is shown. BMI, Body mass index; 6MWD, 6-minutes walking distance; HBCR, home-base cardiac rehabilitation.

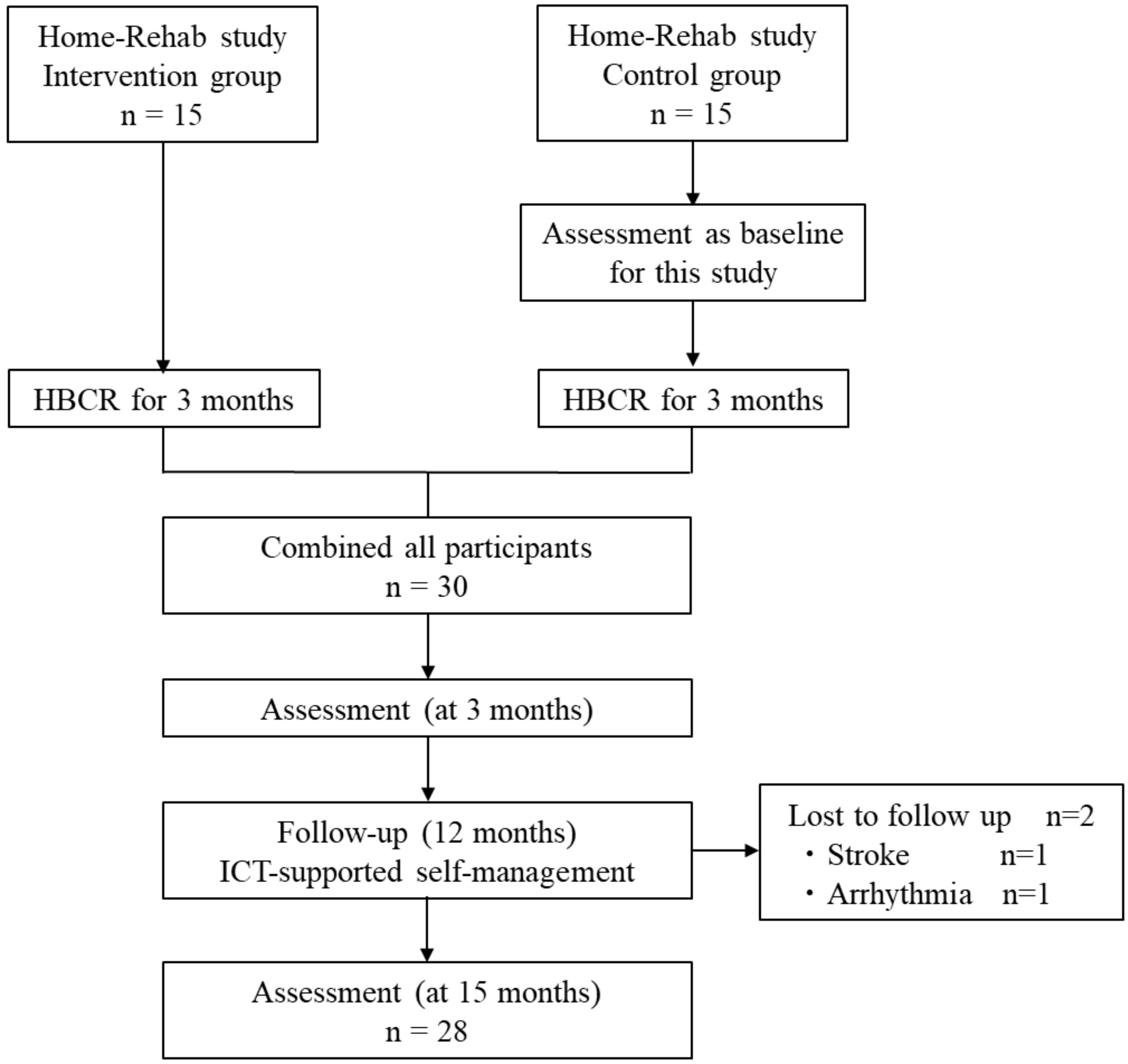

Supplemental Figure S1
